# Retention of patient-held medical records for chronic diseases in Mozambique

**DOI:** 10.11604/pamj.2021.39.1.22504

**Published:** 2021-05-01

**Authors:** Norberto Lumbandali, Ana Mocumbi

**Affiliations:** 1 Instituto Nacional de Saúde, Marracuene District, Mozambique,; 2 Hospital Geral de Mavalane, Maputo, Mozambique,; 3 Universidade Eduardo Mondlane, 3453 Avenida Julius Nyerere, Maputo, Mozambique

**Keywords:** Patient-held medical records, health information, low-income countries

## Abstract

**Introduction:**

Non-Communicable Diseases (NCD) are becoming a public health problem in Mozambique and wider sub-Saharan Africa, and are driving changes to guaranty lifelong follow up of patients within the health systems. Patient-Held Medical Records (PHMR) are an option for this follow-up in under-resourced health systems. We designed a study to assess the rate of retention and quality of conservation of the PHMR.

**Methods:**

we conducted a prospective observational study from November 2016 to October 2018 in a peri-urban hospital from in Mozambique. Consecutive newly diagnosed patients with cardiovascular disease were given PHMR. Data was collected after their first consultation and one year after. The retention and quality of conservation were assessed after 12 months.

**Results:**

overall 134 PHMR were given to patients (24;17.9% children and 77;57.5% female), of which 121 (90.3%) retained at 12 months (90.9% in good conservation state). Most patients had on average four visits to health facilities during the study, all registered in the PHMR. Retention could not be confirmed in 13 patients who did not return the PHMR.

**Conclusion:**

PHMR retention rates were high in an urban low-income setting in Africa, with high quality of conservation, thus supporting its use to replace hospital paper-based medical files. Specific research is recommended on acceptability, quality of information registered and patient´s perception.

## Introduction

Health Information Systems (HIS) in some African countries selectively collect data for surveillance of acute and endemic infectious diseases such as malaria, tuberculosis, diarrheal diseases and HIV/AIDS. This poses a critical challenge to monitor health-related Sustainable Development Goals in the continent because data on morbidity and mortality are incomplete, and often inaccurate and untimely [1]. The dissemination of HIV/AIDS care in the continent has unveiled the need to improve data collection and reporting processes for chronic diseases within these under-resourced health systems. In Mozambique, patient-held medical records (PHMR) are used exclusively for pre-natal and puerperal care, and also for child health care; recently, open medical record systems have been tested for monitoring patient with HIV/AIDS with varying success [2,3].

Functional patient monitoring systems (PMS) are essential to ensure quality in diagnosis and treatment of Non-Communicable Diseases (NCD). Within Mozambique´s public health facilities, paper-based PMS are used, requiring big efforts for archiving as well as for recovering at each hospital visit/admission. This system is associated with frequent loss of information that leads to unnecessary costs of repeating diagnostic procedures. Electronic PMS facilitate facility-level reporting, improve ability to identify patients lost to follow-up, and support both facility and patient management [4] - particularly as NCD become a public health problem [5]. However, in most under-resourced settings in Africa lack of national guidelines and norms for patient level HIS for NCD care, together with limited human capacity and informatics infrastructure, hamper the acquisition of reliable information for patient care and public health policy design [6]. In view of organizing NCD clinics at first referral peri-urban hospitals in Mozambique we designed a study aiming at assessing the retention rates and characterizing the handling of PHMR by patients with chronic diseases being followed as outpatients.

## Methods

We conducted a prospective observational study between November 2016 and October 2018, in a first referral urban hospital with capacity for archiving medical files, in Maputo, Mozambique. To assess the rate of retention at 12 months and the quality of conservation of the PHMR, we selected patients with confirmed diagnosis of cardiovascular disease expected to have chronic follow up in the selected health facility. PHMR were given consecutively to newly diagnosed patients, and demographic and clinical data were collected at time of diagnosis, as well as one year after. Patients were advised to present the PHMR in all contacts with the health system (planned or in emergency), and to ask the assisting health professionals to use it, including taking relevant notes of the clinical appointments. Because the system was being tested, medical records continued to be used for standard patient care, and participants had their follow up without any interference from the research team. At 12 months of the entering the study patients were contacted by telephone to bring their PHMR to the research team.

**PHMR design:** we designed an A5 format (21cm length; 15cm width; 200g) 24-page booklet, with a hard paper cover. It had four sections, namely for: i) personal sociodemographic data; ii) medical history, physical exam, laboratory and other diagnostic evaluation; iii) clinical and biological parameters for follow up; iv) health education. The external appearance, status of the cover and legibility of the content were used to assess quality of PHMR handling or conservation. The conservation was considered: GOOD if the booklet was clean, with all its pages and its content readable; ACCEPTABLE when the content could be easily read but the PHMR appearance was changed, being dirty, wet, partially burnt or with missing the cover page; BAD if the booklet´s information could not be read, there were missing pages, or considerable portions of the pages were burnt or removed. We used double data entry at recruitment and after 12 months follow up: directly on the booklet and in standard case record forms kept by the team as original data sources.

For data analysis we used descriptive statistics. Participants gave written informed consent to participate in the study. The National Bioethics Committee of Mozambique (reference 072/CIBS-INS 2015) approved the study.

## Results

**Population characteristics:** overall 134 PHMR were given to patients. The age of patients varied from 1 month to 90 years; 24 (17.9%) were children and 77 (57.5%) were female. The most common diagnoses were severe and/or complicated hypertension (34; 25%), cardiomyopathy (28; 20.9%) and rheumatic heart disease (15; 11.2%). HIV infection was the commonest comorbidity occurring in 29 (21.6%) patients, 2/3 of whom female (19%; 65.5%). Ten patients died (7.5%). Patient´s characteristics are summarized in Table 1.

**Table 1:** patient´s characteristics and patient-held medical record data

Characteristics		N	%
**Socio-demographic data**		134	
**Gender**	Male	56	41.8
	Female	78	58.2
**Age**	0-14 years	24	17.9
	15-60 years	62	46.3
		
**Patient-Held Medical record data**	> 60 years	48	35.8
**Status assessed**	Yes	117	87.3
	Good	107	91.4
	Acceptable	7	6.0
	Bad	3	2.6
**Lost to follow up**	PHMR not assessed	17	12.9
**Use over 12months**	2	1	0.8
	3	24	27.4
	4	76	65.0
	5	12	17.9
**Clinical data**	>5	4	4.4
**Diagnostic principal**	Arterial Hypertension	34	25.4
	Dilated Cardiomyopathy	26	19.4
	Rheumatic Heart Disease	14	10.4
**Comorbidity**	HIV	25	19
**Mortality**	Death	10	8.5

**Availability of data and material:** datasets used and analyzed during the current study are available from the corresponding author on reasonable request.

**PMHR handling:** none of the invited patients refused to participate, but retention could not be confirmed in 13, who did not return the PMHR and could not be found after one year (9.7%; 10 females). Thus, 121 patients (90.3%) retained the PHMR at 12 months. The quality of conservation of the PHMR was good for 110 (90.9%) patients, acceptable for eight (6.6%) and bad for three (2.5). The median number of contacts with the health services during the 12 months was four. Ten patients died and had their booklet brought to the research team by a next to kin (4 of them were children). All patients followed had medical notes made by the assisting clinicians for all appointments, of which less than 1% (26/507) were illegible.

**Ethical approval and consent to participate:** the study was approved by the National Bioethics Committee in Mozambique (IRB 00002657). Consent to participate was obtained for every participant. For participants under 16 years of age, written informed consent was obtained from a parent or guardian.

## Discussion

High PHMR retention rate was achieved in patients assisted for cardiovascular disease in an under-resourced urban hospital in Mozambique, with a very low proportion of the medical records being damaged and excellent rate of presentation at encounters with health professionals over the 12 months of study. Additionally, the quality of the records done by health professionals was good, thus allowing easy management of the patients by the assisting clinicians if the original medical file could not be found. Our results are relevant for Mozambique and other low-income countries in Africa with an increasing burden of NCD, by suggesting that PMHR may be useful where the health systems are constrained by severe shortage of health workforce, inadequate physical infrastructure to handle archives of paper-based medical files, and low access to digital platforms in health facilities to allow the use electronic medical records.

The high levels of retention with good quality of handling and recording represent an opportunity for our stretched health facilities in Mozambique, which have limited space and health force for managing medical archives. Our results are above those obtained in the neighboring country of Malawi, where 63.9% of 7393 women of reproductive age receiving contraception retained their PHMR at 12 months [7]. Paper-based medical records kept in hospitals may exacerbate the challenges related to patient tracking [8] in under-served settings, as they require extra processes and considerable manpower to ensure timely availability when patients have unexpected visits to the health facility, or even to emergency departments in theirs or in other health facilities.

Digital technology has had a powerful impact on health care delivery in resource-limited settings, including for patient care and tracking [9], and electronic medical records have been shown to improve quality of health care (expectation and perception) [10]. However, they are not yet widespread in Africa.

Because management of risk factors and chronic diseases require long-term follow up and retention of patients in the health system, regardless of change in residency, employment or migration, PHMR are a cost-effective strategy for ensuring quality and continuity of care provided to patients with chronic conditions in the continent. They guaranty prompt access to patient´s medical information, reinforce the relationship between the patient and the health provider [6], and improve the management and control of chronic conditions [11,12]; all these advantages are expected to contribute to improve quality of care and outcomes.

Although widely used for pre-natal and childhood care at peripheral levels of the health system in Mozambique, PHMR have not been the subject of specific research regarding its use, acceptability by users, quality of information registered by health providers or perceptions from any of these groups. PHMR are useful for both the patients and the health professionals, and seem to increase the trust of patients in the health system; they feel themselves as an active part on the management of their health/disease [13]. By improving the patent´s-doctor relationship [6] they may also increase the recording of events on follow up. On the other hand, because they are readily available to the clinicians whenever the patient contacts the health system, they also avoid duplication of procedures, reducing workload and costs. Finally, by allowing rapid access to patient´s clinical information, PHMR improve communication between health providers.

A small proportion of information recorded during the visits could not be read due to bad hand-writing, corroborating expressed concern regarding the quality of information collected by health professionals using PHMR. In South Africa an evaluation of the quality of information content in PHMR used by puerperal women over a six-weeks period, showed that less than 50% of them were correctly filed [11]. This barrier on the health provider´s side needs to be addressed, and may be determined by extreme shortage of personnel in environments with very high demand.

Low or inadequate health literacy in a context of high multimorbidity and patients entering the system through emergency departments [14] contribute to errors and gaps in long-term management as well as to challenges during transitional care. This is particularly evident as patients move between different stages throughout their lifespan (childhood to adolescence, adolescence to adulthood), when they are referred to other levels of the health system (between primary care and specialized hospitals), or whenever they are transferred to different locations. Paper-based PHMR address continuity of care, optimize management and reduce medication errors, a preventable cause of morbidity, disability and mortality.

Our study did not collect data on effect of PHMR on outcomes, and does not allow us to discuss the usefulness of PHMR for health promotion as well as the challenges that patients face regarding confidentiality of records, and therefore qualitative research on the views of users and health professionals is warranted. However, it brings important information on alternative strategies to the current patients monitoring systems for chronic diseases in low-income settings.

## Conclusion

High PHMR retention rates were obtained in an urban low-income setting in Africa, with good quality of conservation and usage by clinicians. This supports its use as an alternative to replace hospital paper-based medical files in under-resourced settings unprepared to deal with the growing demand of chronic care. Qualitative research on the views of health professionals and acceptability by the patients is warranted.

### What is known about this topic

- Functional patient monitoring systems are essential to ensure quality in the diagnosis and treatment of non-communicable diseases;
- The use of facility-based paper patient medical records requires great efforts for archiving and recovery at each visit or admission to the hospital;
- In underserved settings loss of information and unnecessary costs in repeating diagnostic procedures are frequent with the use of facility-based paper medical records.

### What this study adds

- It shows that excellent conservation of patient-held medical records in patients with chronic diseases a low-income setting, as part of their involvement in managing their own disease;
- To improve the control and management of patients with chronic non-communicable diseases patient-held medical records need to be correctly used by health professionals.

## References

[1] Mutale W, Chintu N, Amoroso C, Awoonor-Williams K, Phillips J, Baynes C (2013). Population Health Implementation and Training-Africa Health Initiative Data Collaborative, improving health information systems for decision making across five sub-Saharan African countries: Implementation strategies from the African Health Initiative. BMC Health Serv Res.

[2] Manders EJ, José E, Solis M, Burlison J, Nhampossa JL, Moon T (2010). Implementing OpenMRS for patient monitoring in an HIV/AIDS care and treatment program in rural Mozambique. Stud Health Technol Inform.

[3] Lambdin BH, Micek MA, Koepsell TD, Hughes JP, Sherr K, Pfeiffer J (2012). An assessment of the accuracy and availability of data in electronic patient tracking systems for patients receiving HIV treatment in central Mozambique. BMC Health Serv Res.

[4] Hochgesang M, Zamudio-Haas S, Moran L, Nhampossa L, Packel L, Leslie H (2017). Scaling-up health information systems to improve HIV treatment: An assessment of initial patient monitoring systems in Mozambique. Int J Med Inform.

[5] Silva-Matos C, Beran D (2012). Non-communicable diseases in Mozambique: risk factors, burden, response and outcomes to date. Global Health.

[6] Marutha NS (2017). The role of medical records in the provision of public healthcare services in the Limpopo province of South Africa Research problem. South African Journal of Information.

[7] Dasgupta AN, Ngwalo R, Branson K, Gondwe L, Taulo F, Ngwira B (2015). Using patient-held records to evaluate contraceptive use in Malawi. Bull World Health Organ.

[8] Mkalira Msiska KE, Kumitawa A, Kumwenda B (2017). Factors affecting the utilisation of electronic medical records system in Malawian central hospitals. Malawi Med J.

[9] Gous N, Boeras DI, Cheng B, Takle J, Cunningham B, Peeling RW (2018). The impact of digital technologies on point-of-care diagnostics in resource-limited settings. Expert Rev Mol Diagn.

[10] Ayaad O, Alloubani A, ALhajaa EA, Farhan M, Abuseif S, Al Hroub A (2019). The role of electronic medical records in improving the quality of health care services: Comparative study. Int J Med Inform.

[11] Ramraj T, Goga AE, Larsen A, Ramokolo V, Bhardwaj S, Chirinda W (2018). Completeness of patient-held records: observations of the Road-to-Health Booklet from two national facility-based surveys at 6 weeks postpartum, South Africa. J Glob Health.

[12] Ko H, Turner T, Jones C, Hill C (2010). Patient-held medical records for patients with chronic disease: a systematic review. Qual Saf Health Care.

[13] Codd J (2014). Implementation of a patient-held urinary catheter passport to improve catheter management, by prompting for early removal and enhancing patient compliance. J Infect Prev.

[14] Mocumbi AO, Cebola B, Muloliwa A, Sebastião F, Sitefane SJ, Manafe N (2019). Differential patterns of disease and injury in Mozambique: New perspectives from a pragmatic, multicenter, surveillance study of 7809 emergency presentations. PLoS One.

